# Supplementary material for: CDK5-dependent phosphorylation and nuclear translocation of TRIM59 promotes macroH2A1 ubiquitination and tumorigenicity
Source: Nat Commun. 2019 Sep 5;10:4013. doi: 10.1038/s41467-019-12001-2 (PMC6728346; doi:10.1038/s41467-019-12001-2)
Supplement: Supplementary file 1 — Supplementary Information [file 41467_2019_12001_MOESM1_ESM.pdf]

Supplementary Table 1 GO pathway analysis of mH2A1-bound genes in distance from center of peak (2 kb) in LN229/EGFRvIII cells

	Vehicle control	
Category	Term	FDR
KEGG_PATHWAY	hsa04080:Neuroactive ligand-receptor interaction	1.85E-29
KEGG_PATHWAY	hsa04151:PI3K-Akt signaling pathway	3.15E-28
KEGG_PATHWAY	hsa04024:cAMP signaling pathway	1.50E-17
KEGG_PATHWAY	hsa04015:Rap1 signaling pathway	6.65E-17
KEGG_PATHWAY	hsa05200:Pathways in cancer	4.80E-11
KEGG_PATHWAY	hsa04750:Inflammatory mediator regulation of TRP channels	5.64E-11
KEGG_PATHWAY	hsa04810:Regulation of actin cytoskeleton	9.74E-11
KEGG_PATHWAY	hsa04014:Ras signaling pathway	5.19E-10
KEGG_PATHWAY	hsa04611:Platelet activation	3.10E-09
KEGG_PATHWAY	hsa04060:Cytokine-cytokine receptor interaction	3.49E-08
KEGG_PATHWAY	hsa04630:Jak-STAT signaling pathway	9.91E-08
KEGG_PATHWAY	hsa04510:Focal adhesion	1.63E-07
KEGG_PATHWAY	hsa04020:Calcium signaling pathway	2.79E-07
KEGG_PATHWAY	hsa04713:Circadian entrainment	9.75E-07
KEGG_PATHWAY	hsa04512:ECM-receptor interaction	3.26E-06
KEGG_PATHWAY	hsa04921:Oxytocin signaling pathway	4.49E-06
KEGG_PATHWAY	hsa04022:cGMP-PKG signaling pathway	1.84E-05
KEGG_PATHWAY	hsa04970:Salivary secretion	6.37E-05
KEGG_PATHWAY	hsa04916:Melanogenesis	7.20E-05
KEGG_PATHWAY	hsa05142:Chagas disease (American trypanosomiasis)	6.66E-04
KEGG_PATHWAY	hsa04924:Renin secretion	8.19E-04
KEGG_PATHWAY	hsa04728:Dopaminergic synapse	8.93E-04
KEGG_PATHWAY	hsa05146:Amoebiasis	9.67E-04
KEGG_PATHWAY	hsa04650:Natural killer cell mediated cytotoxicity	0.004046853
KEGG_PATHWAY	hsa05218:Melanoma	0.004288347
KEGG_PATHWAY	hsa04911:Insulin secretion	0.004325345
KEGG_PATHWAY	hsa04972:Pancreatic secretion	0.004997795
KEGG_PATHWAY	hsa04640:Hematopoietic cell lineage	0.006344066
KEGG_PATHWAY	hsa05162:Measles	0.006456921
KEGG_PATHWAY	hsa04730:Long-term depression	0.007414635
KEGG_PATHWAY	hsa04726:Serotonergic synapse	0.008247793
KEGG_PATHWAY	hsa04062:Chemokine signaling pathway	0.009438543
KEGG_PATHWAY	hsa04620:Toll-like receptor signaling pathway	0.012736587
KEGG_PATHWAY	hsa04550:Signaling pathways regulating pluripotency of stem cells	0.017052239
KEGG_PATHWAY	hsa04931:Insulin resistance	0.017449992
KEGG_PATHWAY	hsa04270:Vascular smooth muscle contraction	0.020614883
KEGG_PATHWAY	hsa05152:Tuberculosis	0.024667099
KEGG_PATHWAY	hsa04974:Protein digestion and absorption	0.028504903
KEGG_PATHWAY	hsa04540:Gap junction	0.028504903

KEGG_PATHWAY	hsa04066:HIF-1 signaling pathway	0.030366818
KEGG_PATHWAY	hsa04925:Aldosterone synthesis and secretion	0.0309291
KEGG_PATHWAY	hsa05161:Hepatitis B	0.032491895

Erlotinib treatment		
Category	Term	FDR
KEGG_PATHWAY	hsa04740:Olfactory transduction	1.15E-51
KEGG_PATHWAY	hsa04060:Cytokine-cytokine receptor interaction	9.34E-20
KEGG_PATHWAY	hsa04080:Neuroactive ligand-receptor interaction	1.44E-13
KEGG_PATHWAY	hsa04630:Jak-STAT signaling pathway	1.44E-13
KEGG_PATHWAY	hsa05160:Hepatitis C	4.02E-08
KEGG_PATHWAY	hsa05032:Morphine addiction	1.02E-07
KEGG_PATHWAY	hsa04151:PI3K-Akt signaling pathway	1.43E-07
KEGG_PATHWAY	hsa04973:Carbohydrate digestion and absorption	6.58E-07
KEGG_PATHWAY	hsa04062:Chemokine signaling pathway	9.91E-07
KEGG_PATHWAY	hsa04024:cAMP signaling pathway	2.50E-06
KEGG_PATHWAY	hsa05230:Central carbon metabolism in cancer	1.48E-05
KEGG_PATHWAY	hsa04620:Toll-like receptor signaling pathway	1.09E-04
KEGG_PATHWAY	hsa05162:Measles	1.43E-04
KEGG_PATHWAY	hsa05161:Hepatitis B	3.68E-04
KEGG_PATHWAY	hsa05200:Pathways in cancer	5.02E-04
KEGG_PATHWAY	hsa04650:Natural killer cell mediated cytotoxicity	0.002476769
KEGG_PATHWAY	hsa05033:Nicotine addiction	0.005561919
KEGG_PATHWAY	hsa04723:Retrograde endocannabinoid signaling	0.007079329
KEGG_PATHWAY	hsa04380:Osteoclast differentiation	0.010791786
KEGG_PATHWAY	hsa04727:GABAergic synapse	0.014314665
KEGG_PATHWAY	hsa04514:Cell adhesion molecules (CAMs)	0.0189606
KEGG_PATHWAY	hsa04660:T cell receptor signaling pathway	0.019722166
KEGG_PATHWAY	hsa04724:Glutamatergic synapse	0.020545074
KEGG_PATHWAY	hsa04014:Ras signaling pathway	0.021988436
KEGG_PATHWAY	hsa04725:Cholinergic synapse	0.038832011
KEGG_PATHWAY	hsa04917:Prolactin signaling pathway	0.041610124
KEGG_PATHWAY	hsa04022:cGMP-PKG signaling pathway	0.042262923

Roscovitine treatment		
Category	Term	FDR
KEGG_PATHWAY	hsa04080:Neuroactive ligand-receptor interaction	1.29E-35
KEGG_PATHWAY	hsa04024:cAMP signaling pathway	3.01E-16
KEGG_PATHWAY	hsa04151:PI3K-Akt signaling pathway	6.03E-15
KEGG_PATHWAY	hsa04014:Ras signaling pathway	3.24E-10
KEGG_PATHWAY	hsa04015:Rap1 signaling pathway	2.53E-09
KEGG_PATHWAY	hsa04020:Calcium signaling pathway	7.99E-09
KEGG_PATHWAY	hsa04724:Glutamatergic synapse	2.25E-08

KEGG_PATHWAY	hsa04630:Jak-STAT signaling pathway	6.37E-08
KEGG_PATHWAY	hsa05032:Morphine addiction	1.17E-06
KEGG_PATHWAY	hsa05033:Nicotine addiction	7.75E-05
KEGG_PATHWAY	hsa04010:MAPK signaling pathway	1.22E-04
KEGG_PATHWAY	hsa05030:Cocaine addiction	1.49E-04
KEGG_PATHWAY	hsa04022:cGMP-PKG signaling pathway	1.91E-04
KEGG_PATHWAY	hsa04950:Maturity onset diabetes of the young	4.43E-04
KEGG_PATHWAY	hsa04060:Cytokine-cytokine receptor interaction	4.51E-04
KEGG_PATHWAY	hsa04810:Regulation of actin cytoskeleton	5.23E-04
KEGG_PATHWAY	hsa04921:Oxytocin signaling pathway	9.92E-04
KEGG_PATHWAY	hsa04640:Hematopoietic cell lineage	0.002553
KEGG_PATHWAY	hsa04713:Circadian entrainment	0.008991
KEGG_PATHWAY	hsa04728:Dopaminergic synapse	0.012143
KEGG_PATHWAY	hsa04550:Signaling pathways regulating pluripotency of stem cells	0.013973
KEGG_PATHWAY	hsa05200:Pathways in cancer	0.018766
KEGG_PATHWAY	hsa05031:Amphetamine addiction	0.03787
KEGG_PATHWAY	hsa04261:Adrenergic signaling in cardiomyocytes	0.037945
KEGG_PATHWAY	hsa04970:Salivary secretion	0.042805

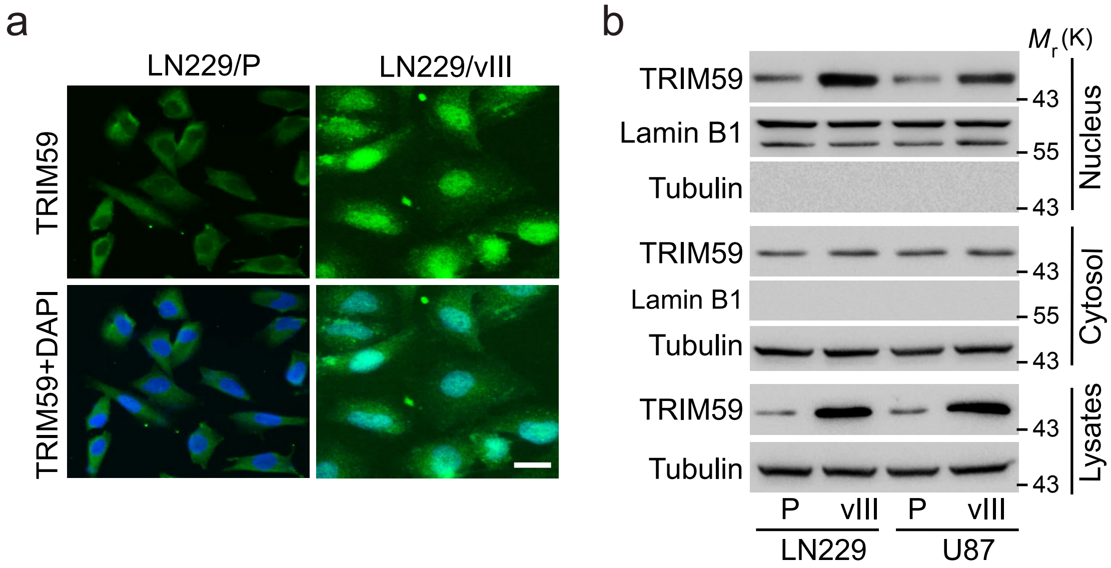

Supplementary Figure 1 Effect of EGF stimulation on TRIM59 protein stability

a. IF analysis of TRIM59 nuclear translocation in LN229 GBM cells with or without EGFRvIII expression. P, parental. vIII, EGFRvIII. Scale bars, 40 μM.

b. WB for TRIM59 in LN229 and U87 GBM cells with or without EGFRvIII expression.

Data are representative of three independent experiments with similar results.

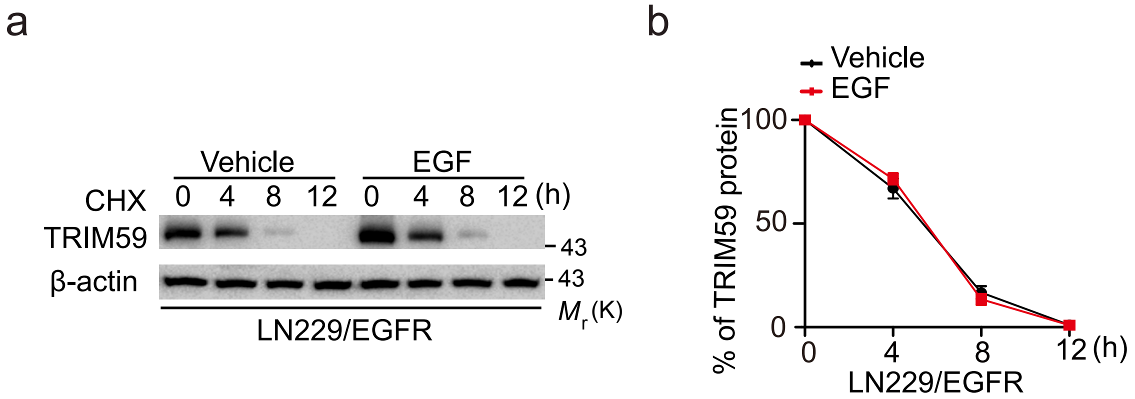

Supplementary Figure 2 Effect of EGF stimulation on TRIM59 protein stability

a. WB assays of effects of EGF treatment on TRIM59 protein levels in LN229/EGFR cells. After stimulation with EGF (100 ng/ml) for 6 h, cells were treated with cycloheximide (CHX, 20 mg/ml) for the indicated time points.

b. Quantification of TRIM59 protein levels in a.

Data are representative of three independent experiments with similar results. Source data are provided as a Source Data file.

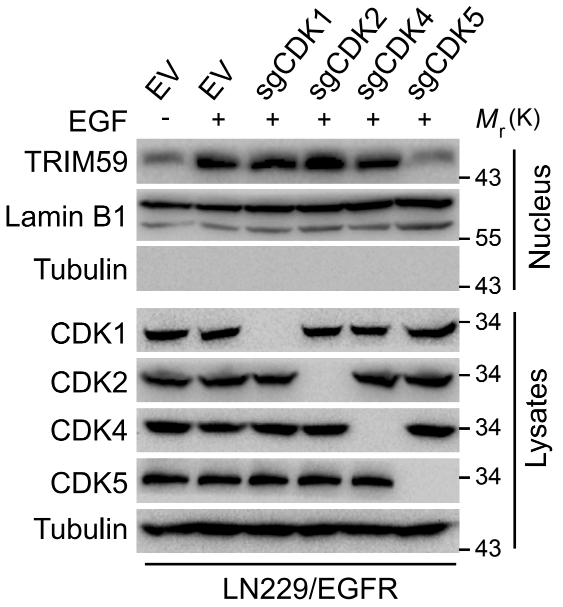

Supplementary Figure 3 Effects of CDK1, CDK2, CDK4 or CDK5 knockout on TRIM59 nuclear localization

WB assays of effects of CDKs knockout on TRIM59 nuclear translocation in LN229/EGFR cells with or without EGF (100 ng/ml) stimulation for 6 h. Data are representative of three independent experiments with similar results.

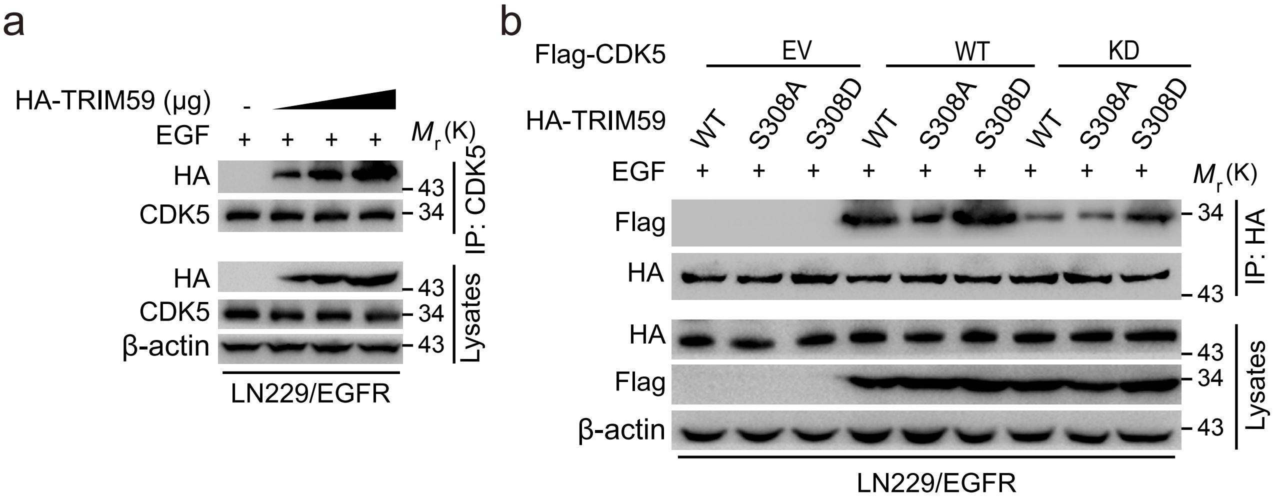

Supplementary Figure 4 Effects of elevated TRIM59 expression or CDK5 kinase activity on CDK5-TRIM59 interaction

a. IP and WB for TRIM59 binding to CDK5 in LN229/EGFR cells transfected with increasing amounts of TRIM59 with EGF stimulation.

b. Effects of ectopic expression of TRIM59 WT, S308A, or S308D mutant on TRIM59 association with CDK5 WT or KD mutant with EGF stimulation.

Data are representative of three independent experiments with similar results.

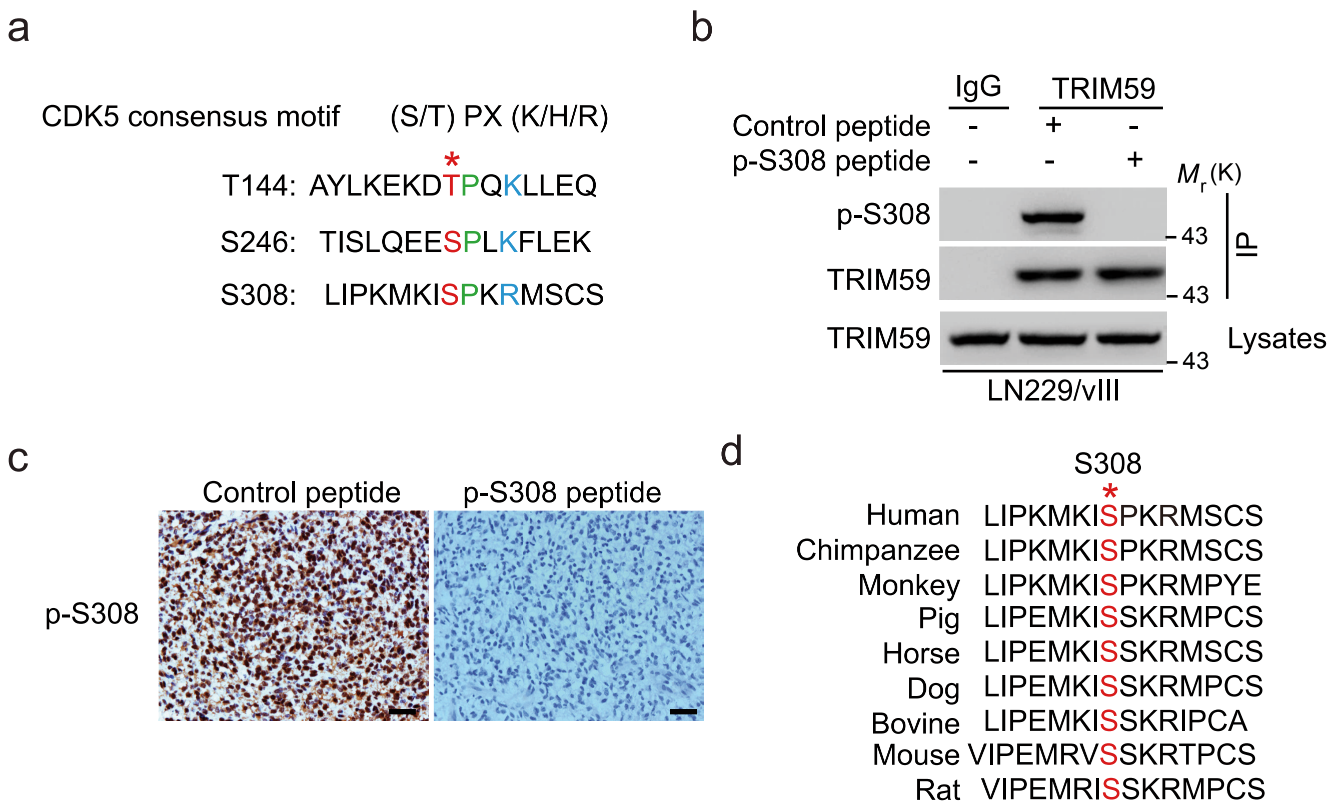

Supplementary Figure 5 Validation of the specificity of the anti-p-TRIM59^{S308} antibody

a. The amino acid sequences around the sites potentially targeted by CDK5 in TRIM59.
b. IP and WB for TRIM59 phosphorylation in LN229/EGFRvIII GBM cells. A rabbit p-TRIM59^{S308} was generated against a specific phospho-peptide containing p-S308. Before IP, agarose beads were pre-incubated with a control peptide or the specific phospho-peptide containing p-S308.
c. IHC assays of a clinical GBM tumor tissue with the specific anti-p-TRIM59^{S308} antibody in the presence of a control peptide or the specific phospho-peptide containing p-S308. IHC was performed twice on the GBM sample with the blocking peptide with similar results. Scale bar, 50μm.
d. Amino acid sequences around S308 in TRIM59 among multiple species.

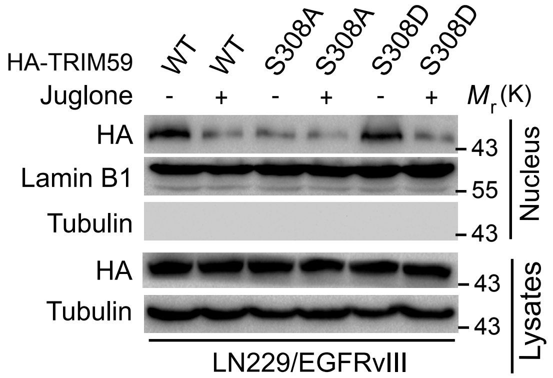

Supplementary Figure 6 Effect of PIN1 inhibitor on TRIM59 nuclear translocation

LN229/EGFRvIII GBM cells reconstituted with TRIM59 WT, S308A or S308D mutant were treated with or without PIN1 inhibitor Juglone (10 μM) for 4 h. Data are representative of three independent experiments with similar results.

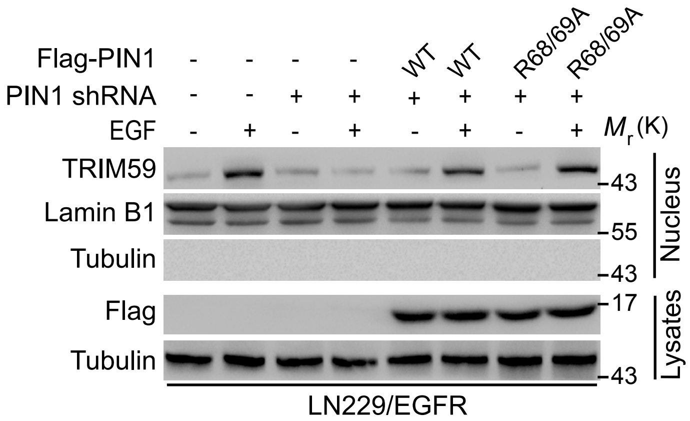

Supplementary Figure 7 Effect of re-expression of PIN1 WT and R68/69A mutant on TRIM59 nuclear translocation

LN229/EGFRvIII GBM cells reconstituted with empty vector (EV), shRNA resistant PIN1 WT, or R68/69A mutant were stimulated with or without EGF (100 ng/ml) for 6 h. Data are representative of three independent experiments with similar results.

Supplementary Figure 8 Identification of macroH2A1 as a TRIM59 binding partner by LC-MS/MS analysis. Peptide sequences of macroH2A1 with seven independent tryptic peptides identified by LC-MS/MS analysis.

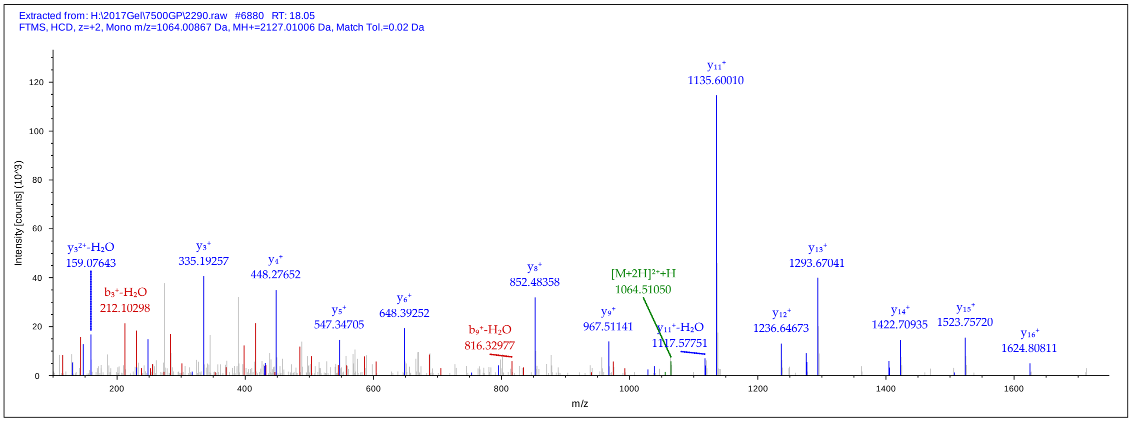

Sequence #1: AASADSTTEGTPADGFTVLSTK

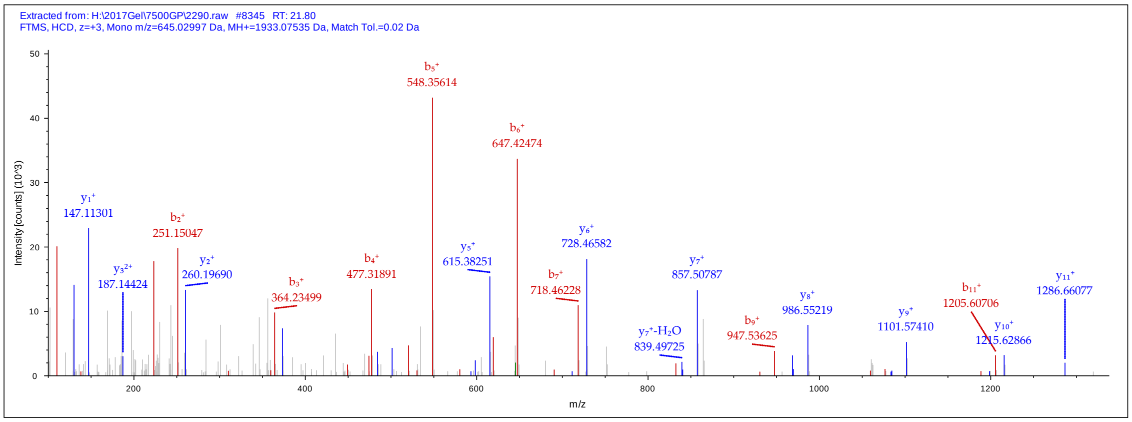

Sequence #2: HILLAVANDEELNQLLK

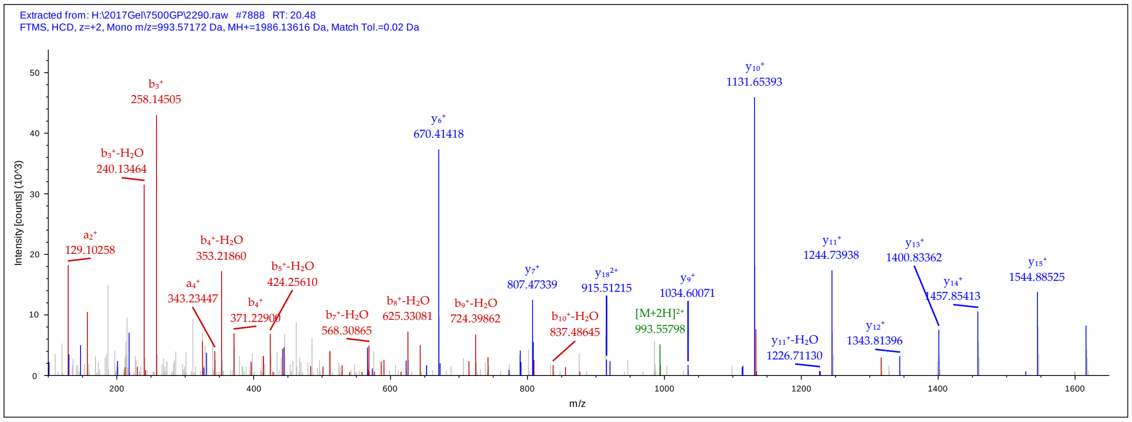

Sequence #3: GVTIASGGVLPNIHPELLAK

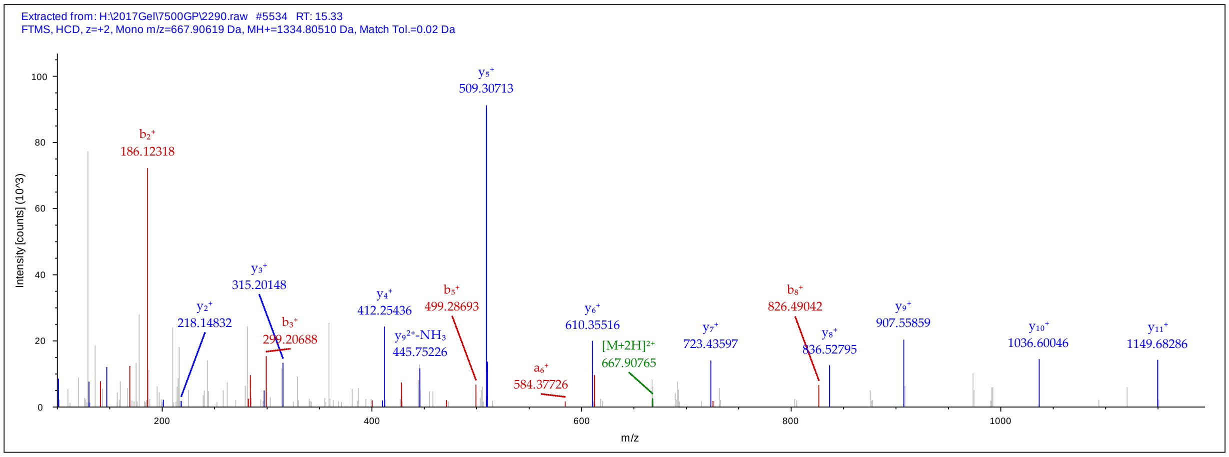

Sequence #4: GKLEAIITPPPAK

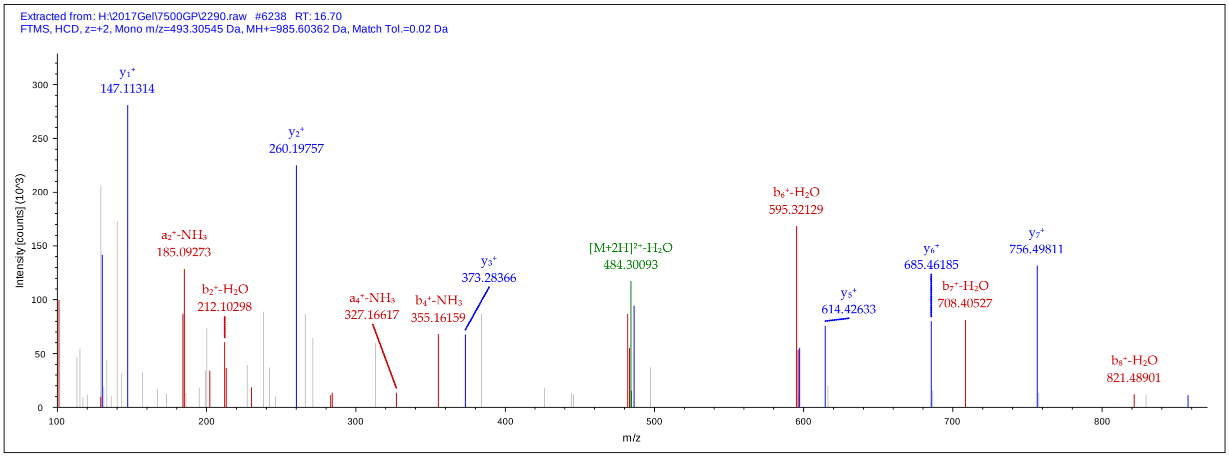

Sequence #5: QTAAQLILK

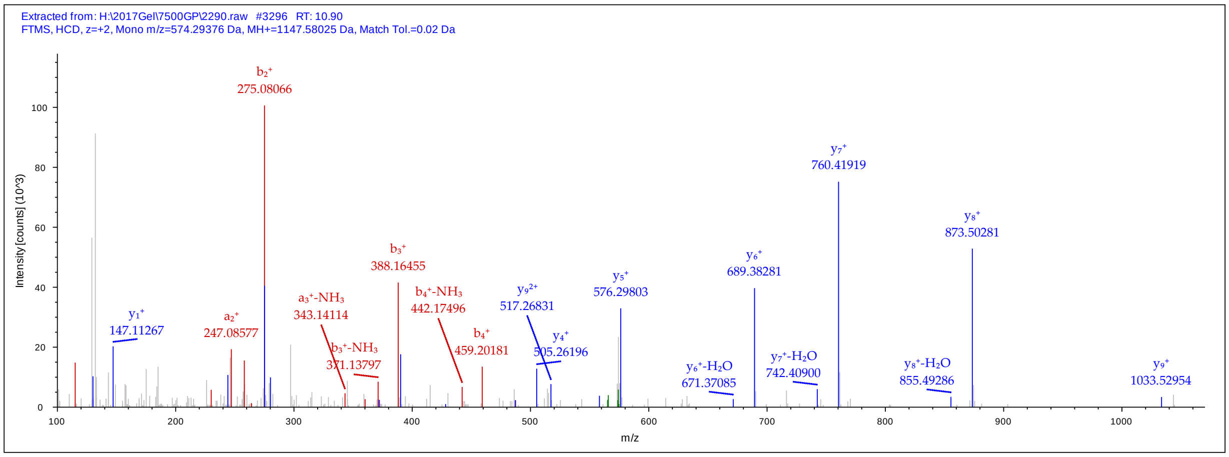

Sequence #6: NCLALADDKK

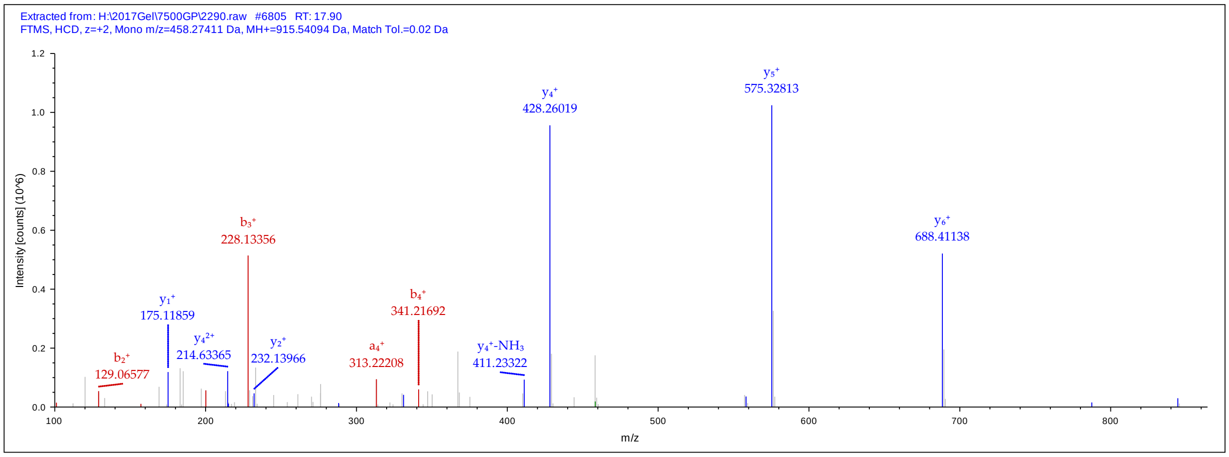

Sequence #7: AGVIFPVGR

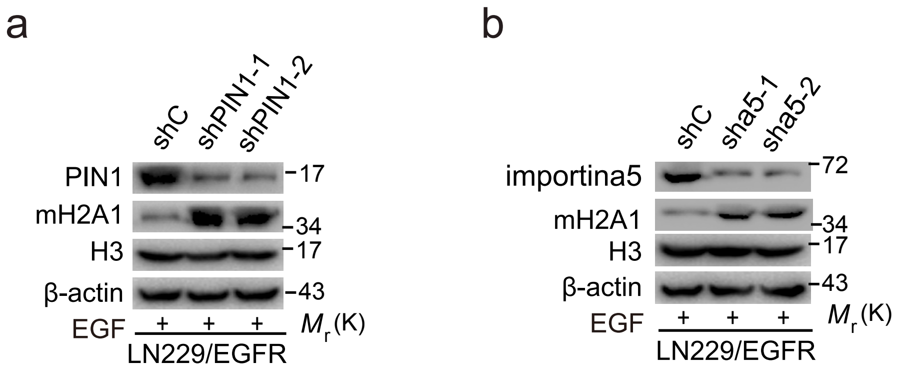

Supplementary Figure 9 Effects of PIN1 or importin α5 knockdown on the level of mH2A1 protein

a and b. WB assay of effects of PIN1 or importin α5 knockdown on the level of mH2A1 protein in LN229/EGFR GBM cells with EGF stimulation. Data are representative of three independent experiments with similar results.

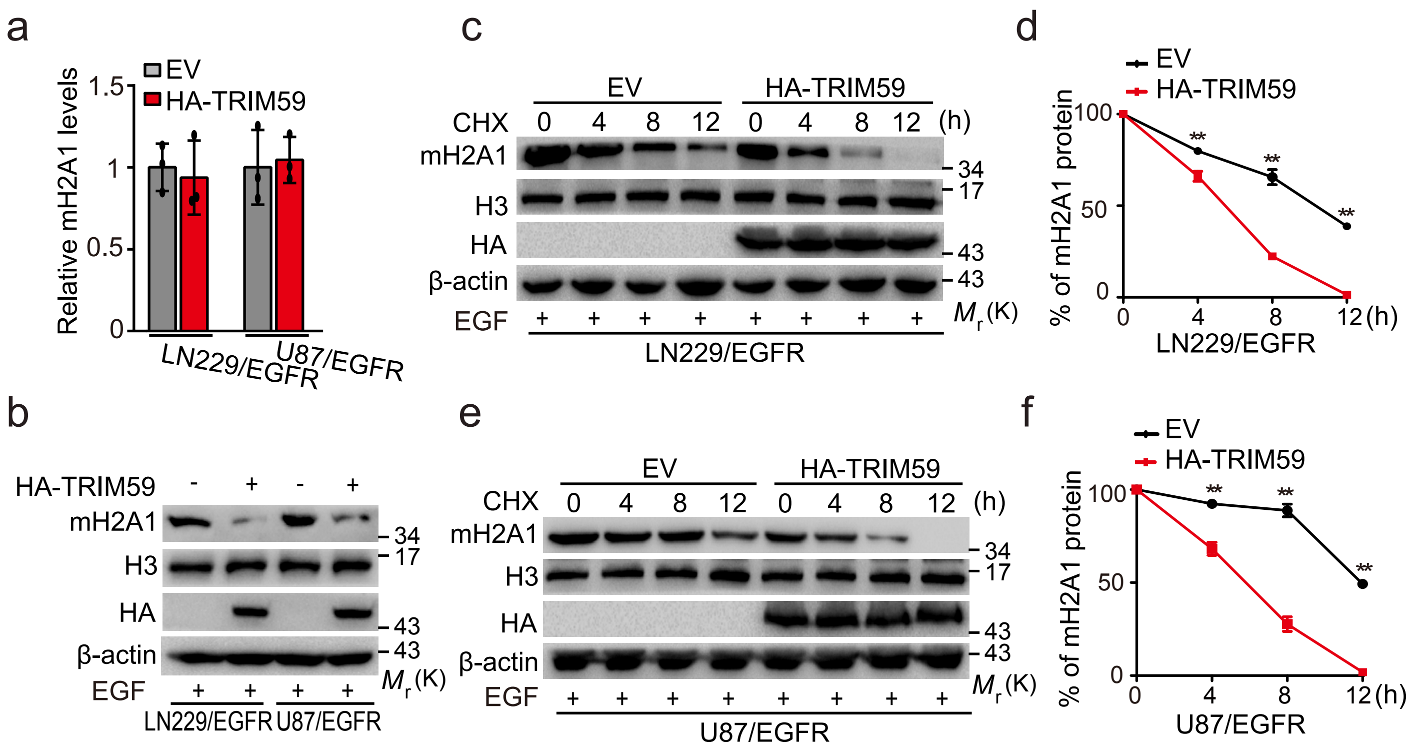

Supplementary Figure 10 Ectopic expression of TRIM59 promotes macroH2A1 degradation

a and b. qRT-PCR and WB assays of effects of TRIM59 overexpression on macroH2A1 mRNA (a) and protein (b) levels in LN229/EGFR and U87/EGFR cells stimulated with EGF.

c and e. Effects of TRIM59 overexpression on macroH2A1 (mH2A1) stability in LN229/EGFR (c) and U87/EGFR (e) cells. After stimulation with EGF (100 ng/ml) for 6 h, cells were treated with cycloheximide (CHX, 20 mg/ml) for the indicated time points.

d and f. Quantification of mH2A1 protein levels in c and e, respectively.

Data are representative of three independent experiments with similar results. Data were expressed as means ± SD. $^{**}P < 0.01$, by two-tailed Student's *t* test. Source data are provided as a Source Data file.

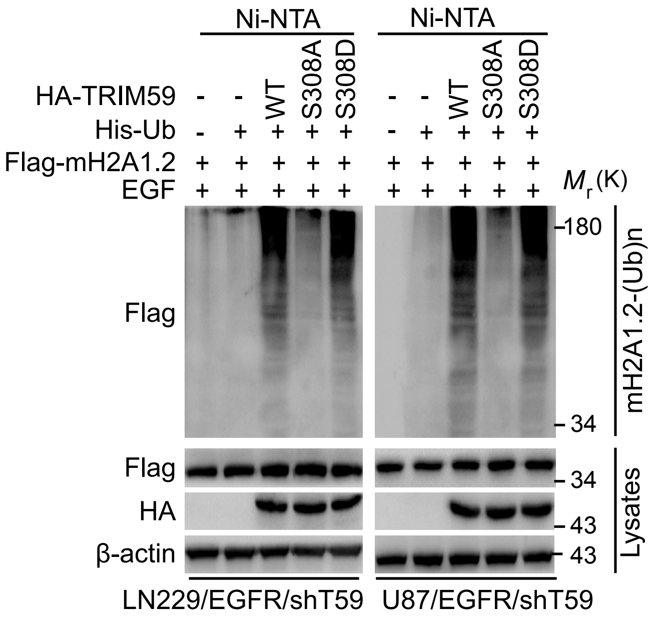

Supplementary Figure 11 Effects of TRIM59 WT, S308A, or S308D mutant on mH2A1.2 ubiquitination

Ubiquitination analysis. His-tagged ubiquitin (His-Ub) was co-transfected into LN229/EGFR/shTRIM59 and U87/EGFR/shTRIM59 cells with TRIM59 constructs or empty vector control. Various cells were stimulated with EGF (100ng/ml) for 6h. Data are representative of two independent experiments with similar results.

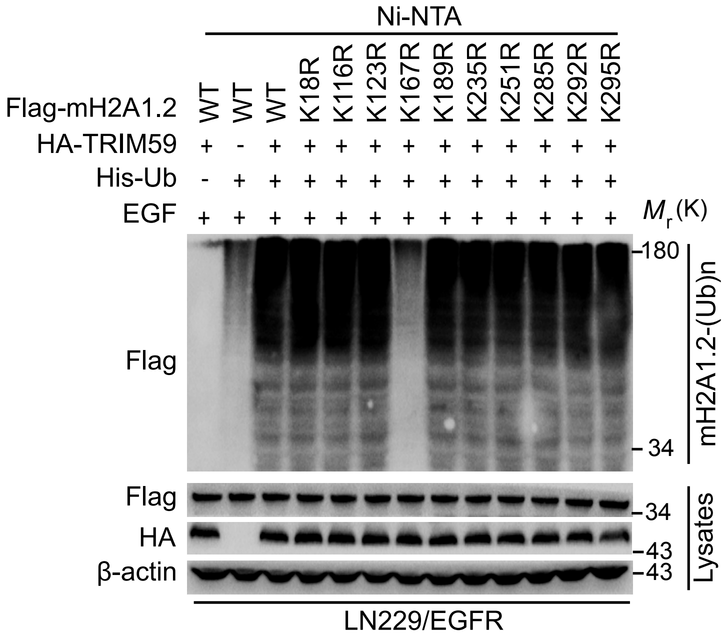

Supplementary Figure 12 Effects of mH2A1 indicated ubiquitin mutants on mH2A1 ubiquitination by TRIM59

His-tagged ubiquitin (His-Ub) was co-transfected into LN229/EGFR cells with TRIM59 and mH2A1 WT or K-to-R mutants with EGF (100ng/ml) stimulation for 6h. Data are representative of three independent experiments with similar results.

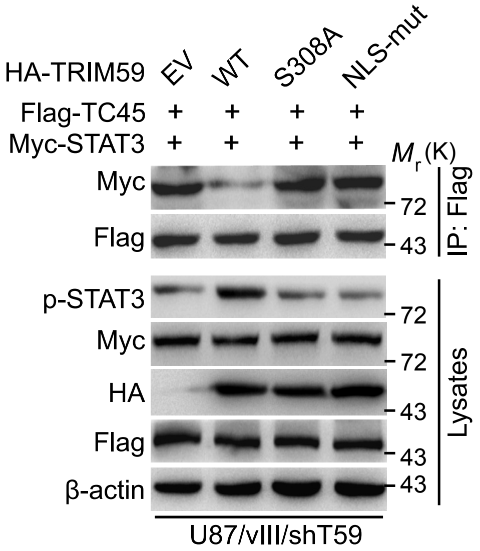

Supplementary Figure 13 Effects of TRIM59, S308A, or NLS-mut mutant on the association between STAT3 and TC45

IP and WB assay of effects of TRIM59 WT, S308A, or NLS-mut mutant on the association between STAT3 and TC45. Myc-tagged STAT3 was co-transfected with or without Flag-TC45, HA-TRIM59 WT, S308A, or NLS-mut mutant into U87/EGFRvIII GBM cells. Data are representative of three independent experiments with similar results.

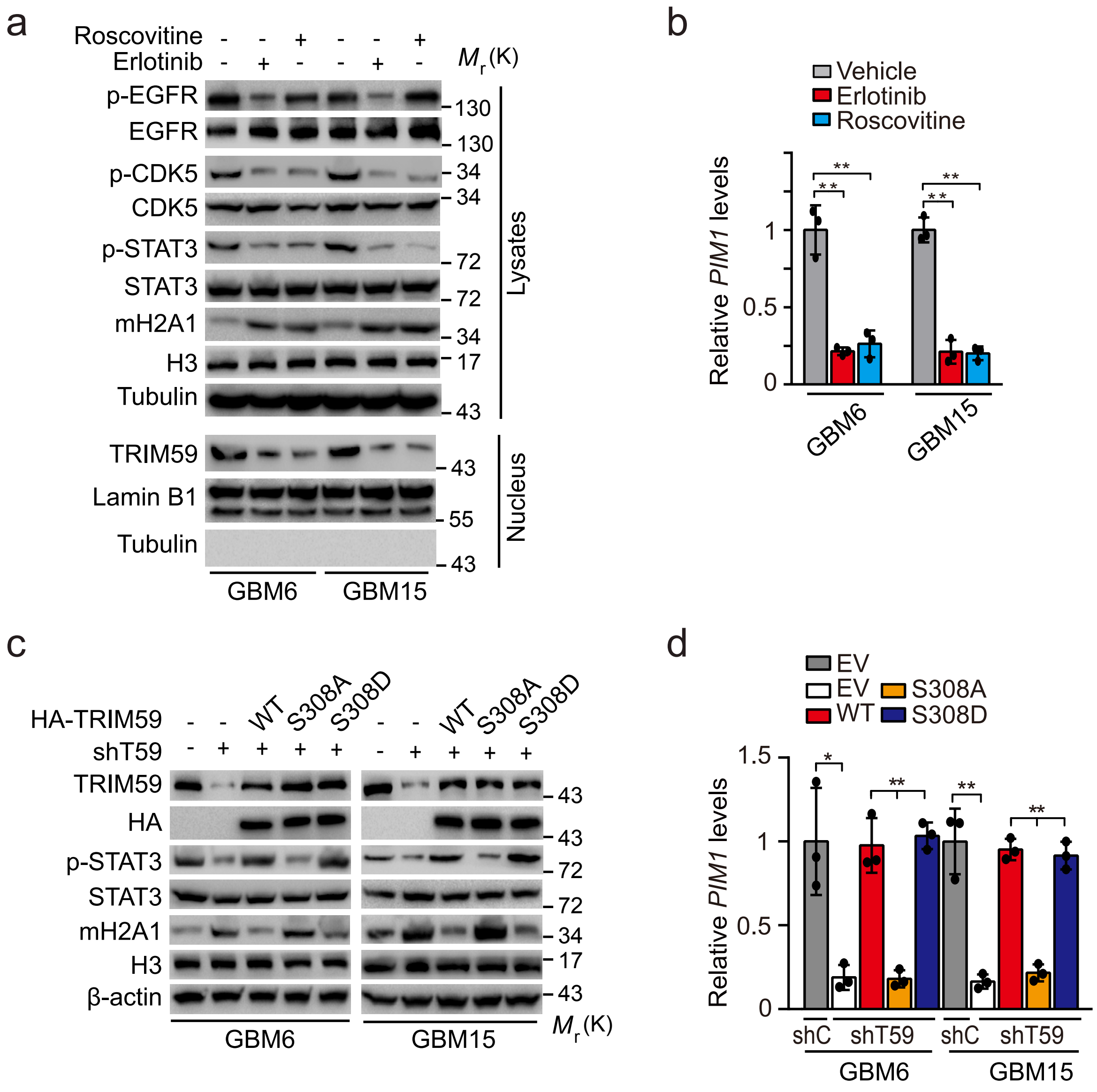

Supplementary Figure 14 CDK5-dependent TRIM59 phosphorylation regulates STAT3 activity in patient-derived primary GBM cells

a. Effects of EGFR inhibitor erlotinib or CDK5 inhibitor Roscovitine treatment on p-EGFR, p-CDK5, p-STAT3, and mH2A1 protein levels in GBM6 and GBM15 cells.

b. qRT-PCR analyses of erlotinib or Roscovitine treatment on STAT3 target gene *PIM1* mRNA expression.

c-d. Effects of TRIM59 shRNA knockdown and re-expression of shRNA-resistant TRIM59 WT, S308A, or S308D mutant on TRIM59, mH2A1 protein levels (c), p-STAT3 (c), STAT3 target gene *PIM1* expression (d) in GBM6 and GBM15 cells.

Data are representative of three independent experiments with similar results. Data were expressed as means ± SD. **P* < 0.05, ***P* < 0.01, by two-tailed Student's *t* test. Source data are provided as a Source Data file.

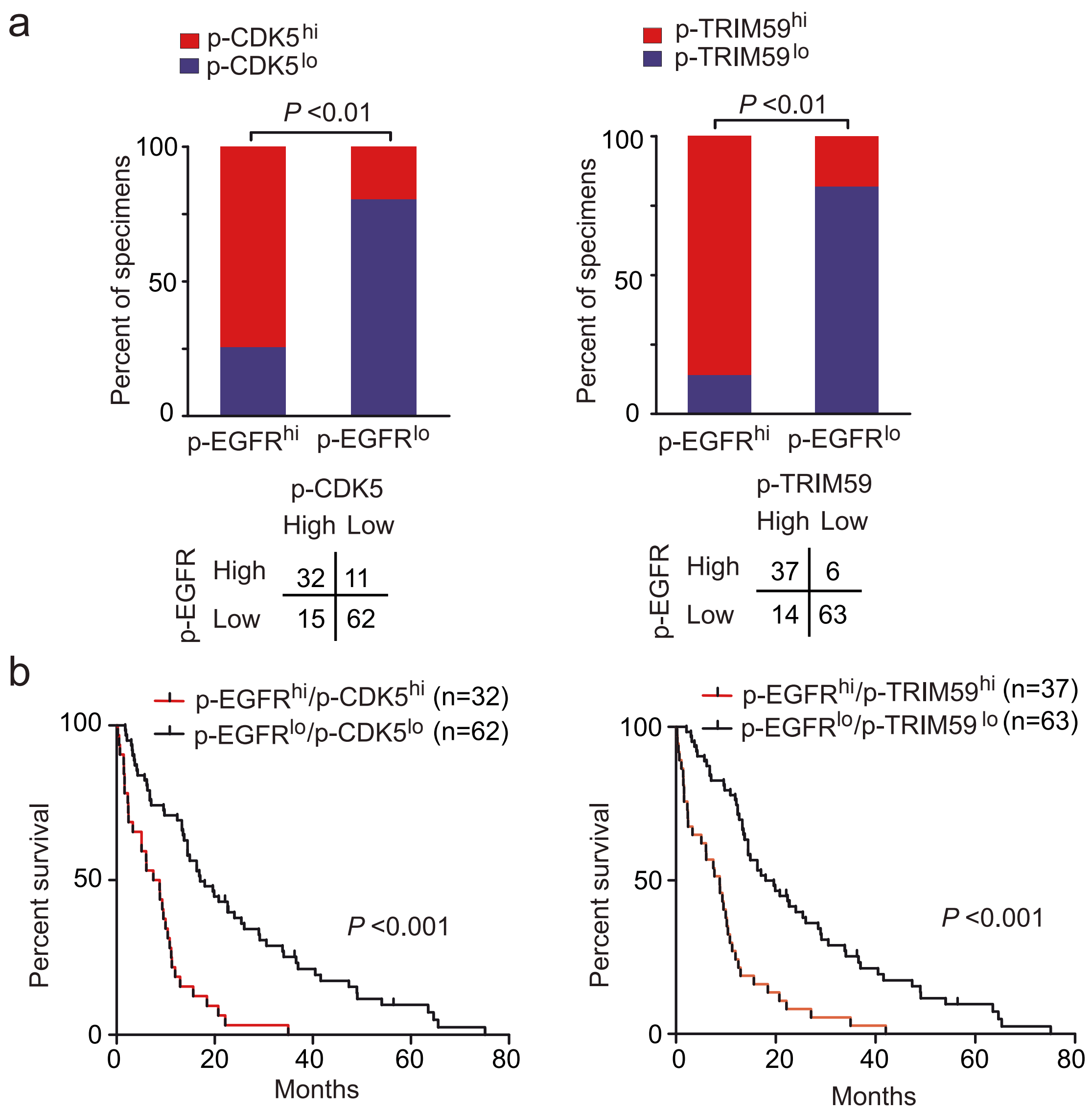

Supplementary Figure 15 Levels of p-EGFR correlate with p-CDK5 and p-TRIM59^{S308} in clinical GBM specimens

a. Correlation of expression between p-EGFR^{Y1173} with p-CDK5^{Y15} and p-TRIM59^{S308} in Figure 8A.

b. Kaplan-Meier survival analysis of GBM patients with tumors expressing indicated proteins. Median survival (in months): p-EGFR^{hi}/p-CDK5^{hi}, 8.12; p-EGFR^{lo}/p-CDK5^{lo}, 17.03; p-EGFR^{hi}/p-TRIM59^{hi}, 8.77; p-EGFR^{lo}/p-TRIM59^{lo}, 17.97. Statistical analysis was performed by log-rank test. Source data are provided as a Source Data file.

Figure 1B

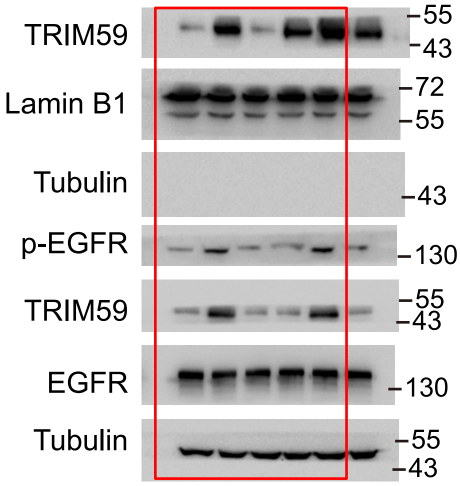

Figure 1C

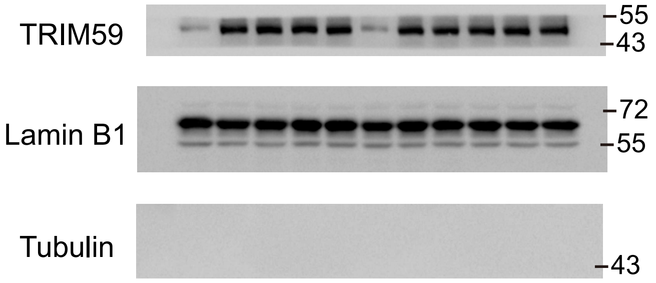

Figure 1E

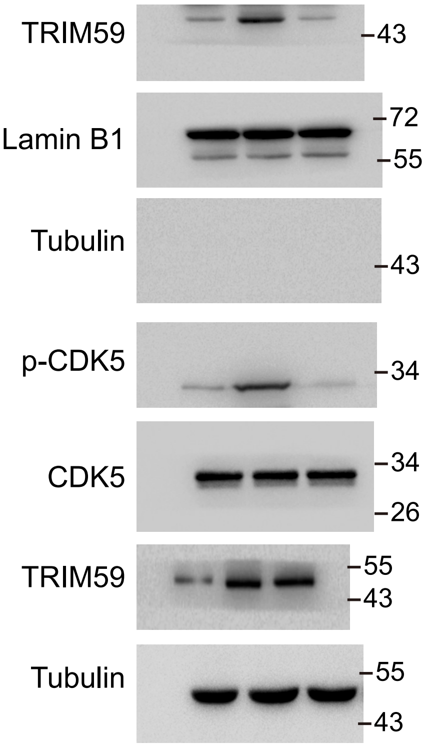

Figure 1F

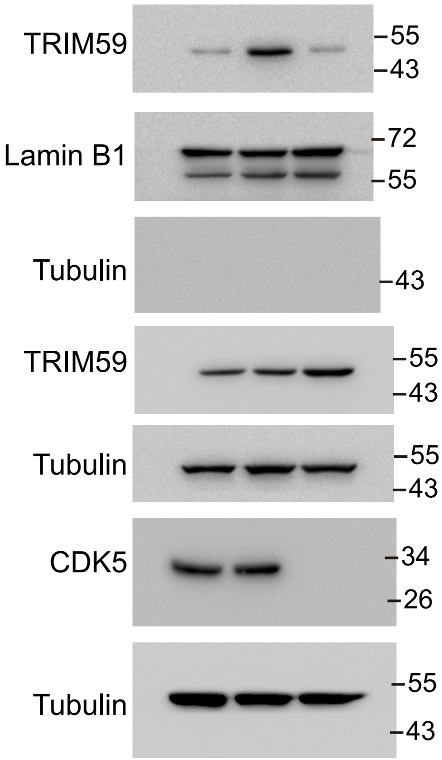

Figure 1G

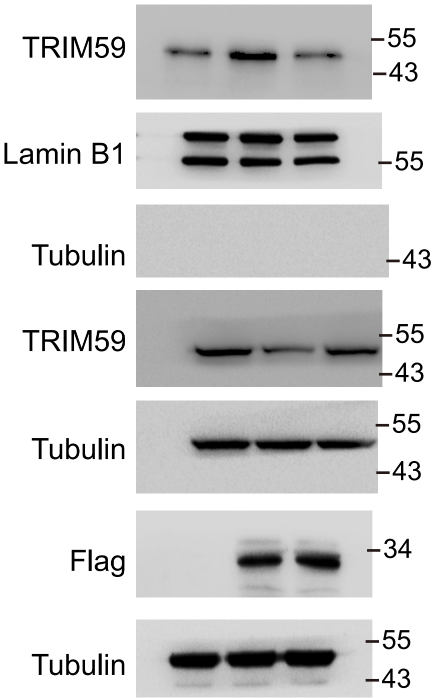

Figure 2A

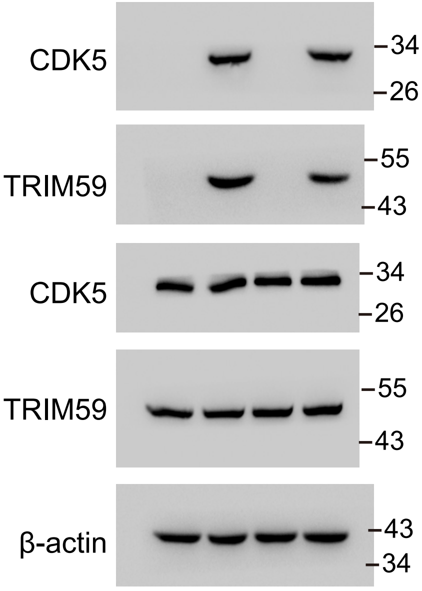

Figure 2B

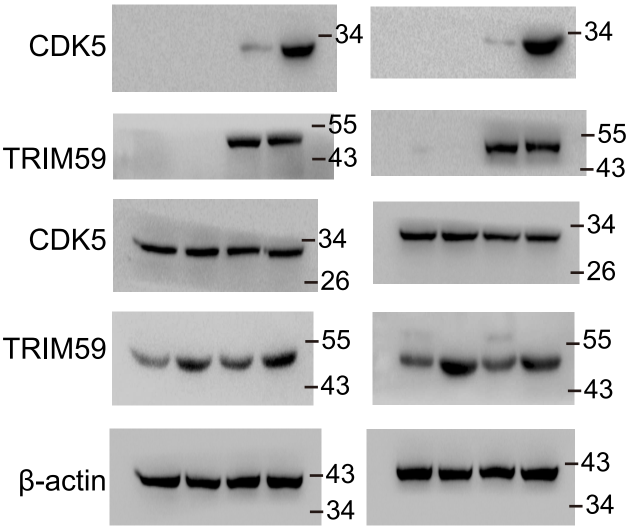

Figure 2C

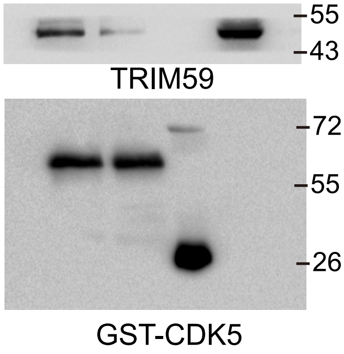

Figure 2E

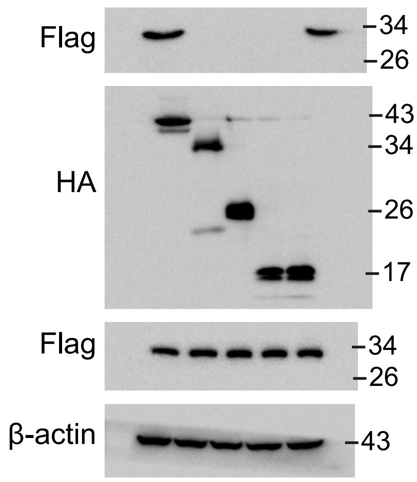

Figure 2F

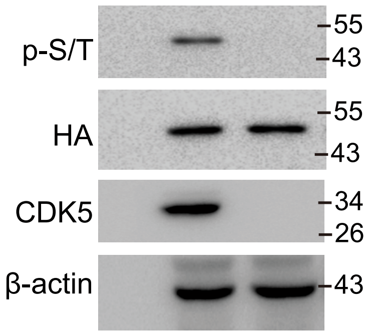

Figure 2G

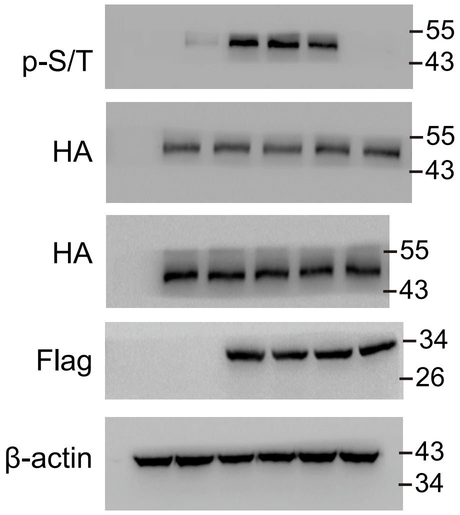

Figure 2H

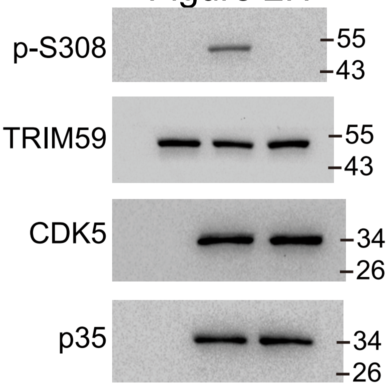

Figure 2I

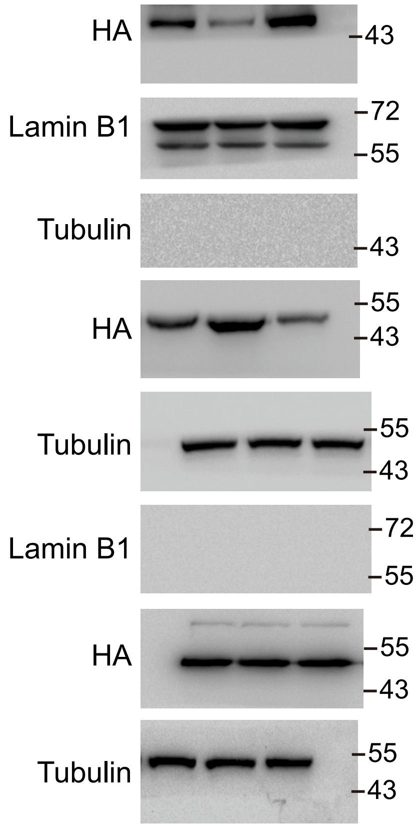

Figure 3A

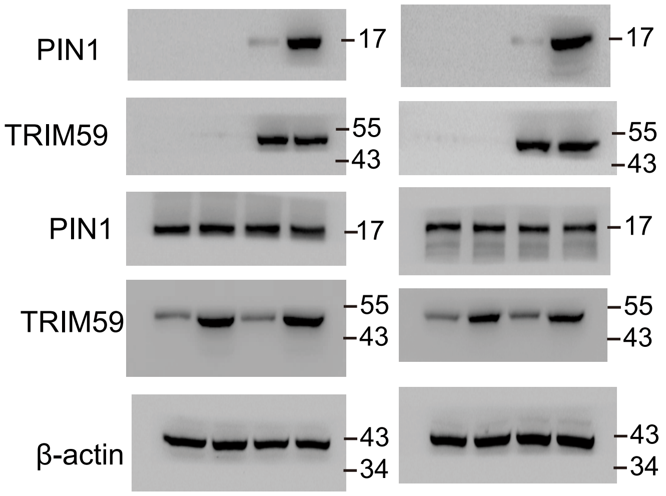

Figure 3B

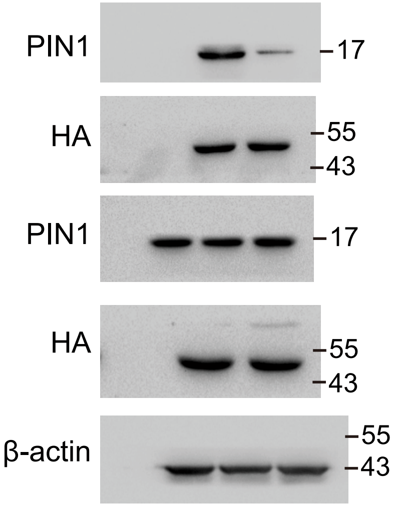

Figure 3C

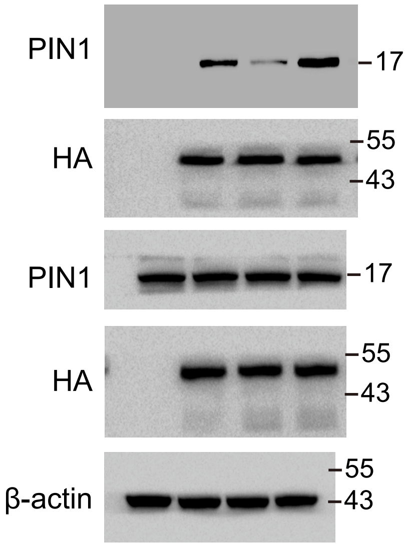

Figure 3D

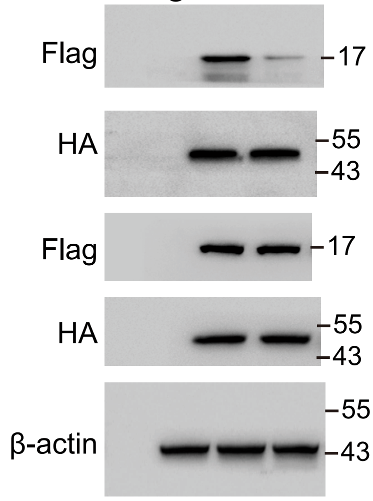

Figure 3F

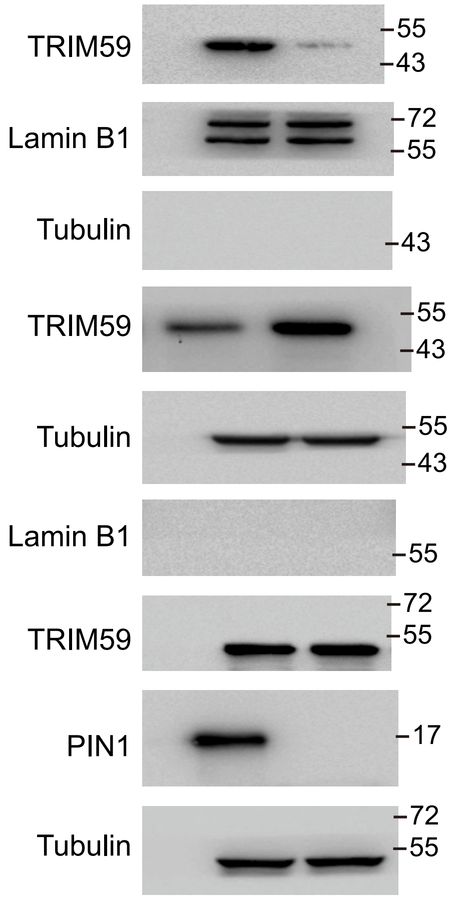

Figure 3G

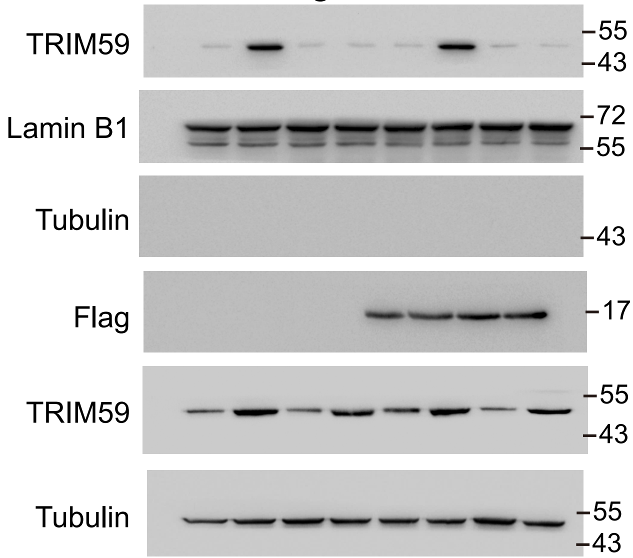

Figure 4C

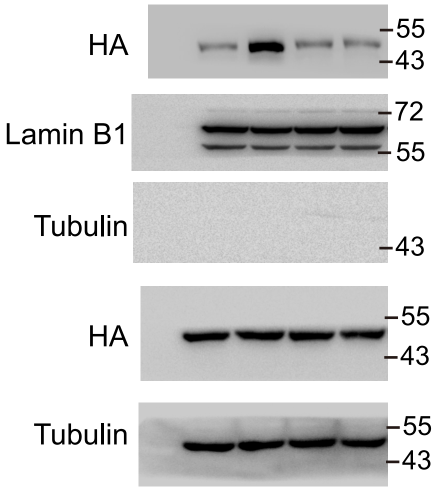

Figure 4D

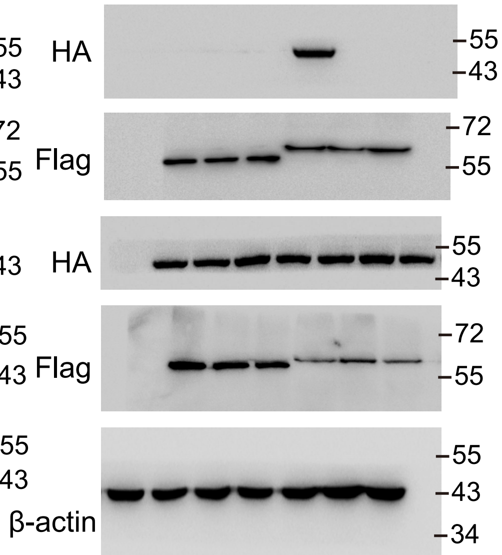

Figure 4E

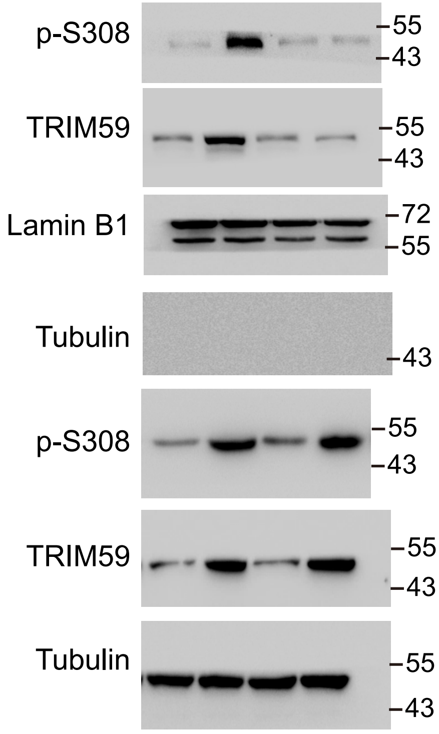

Figure 4F

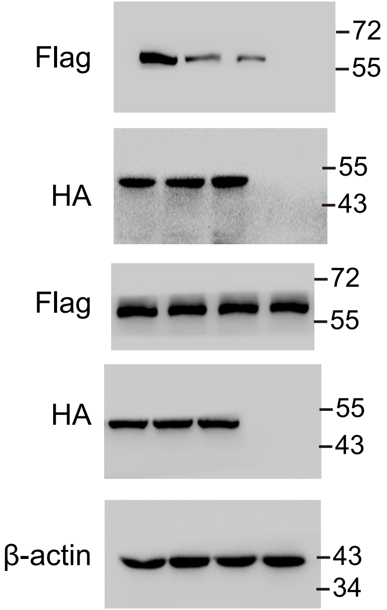

Figure 4G

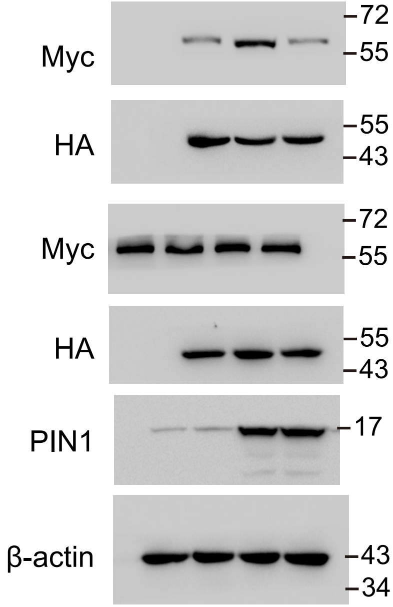

Figure 5B

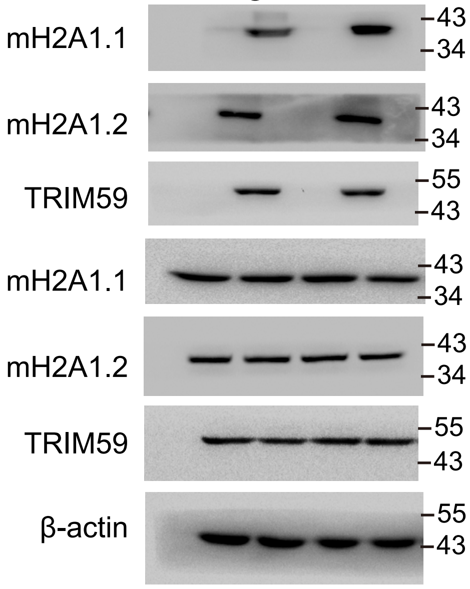

Figure 5D

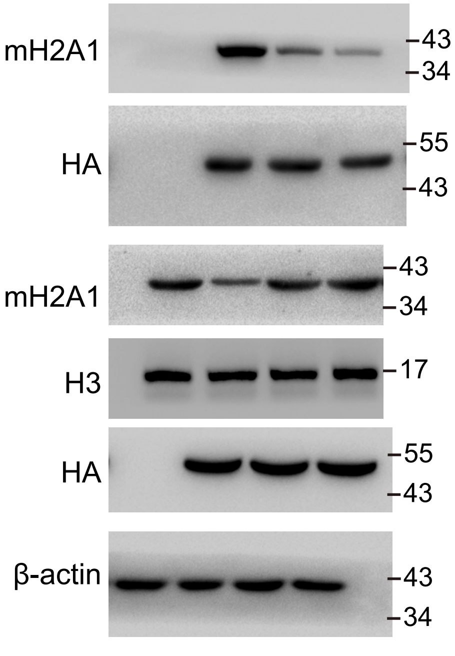

Figure 6A

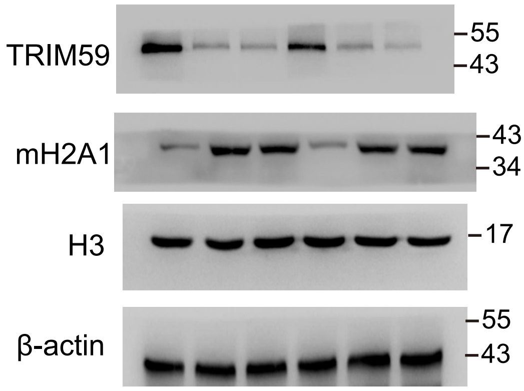

Figure 6C

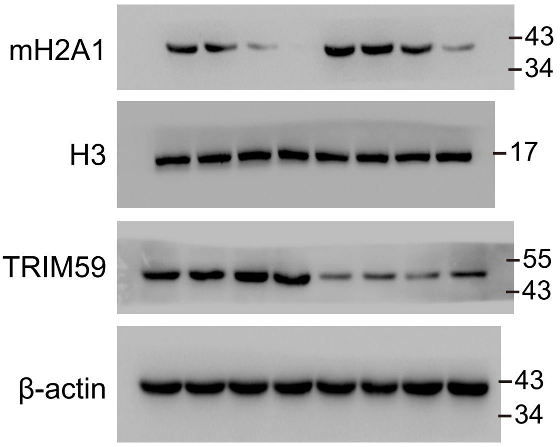

Figure 6E

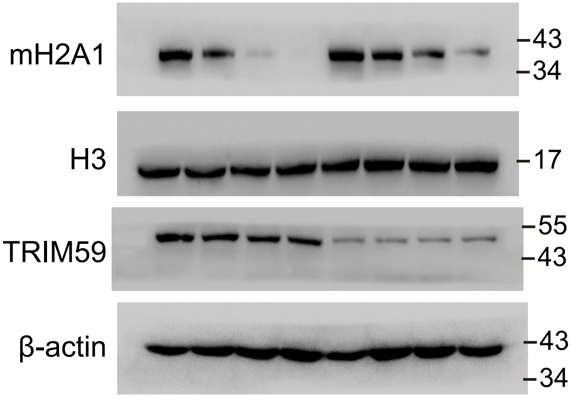

Figure 6I

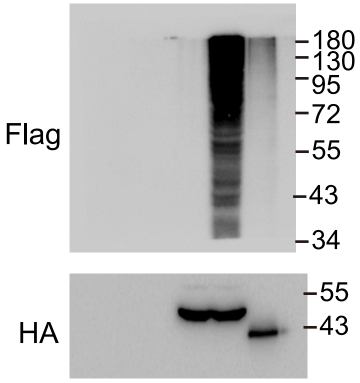

Figure 6G

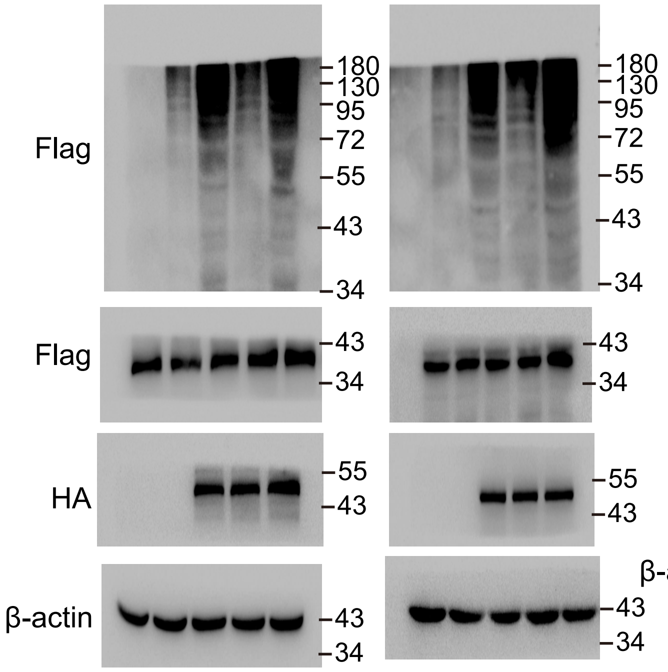

Figure 6H

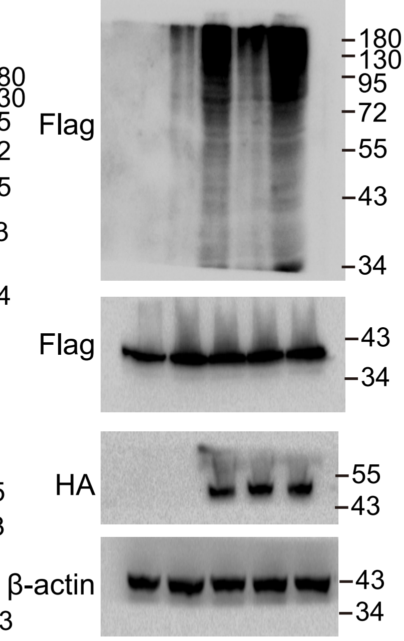

Figure 7F

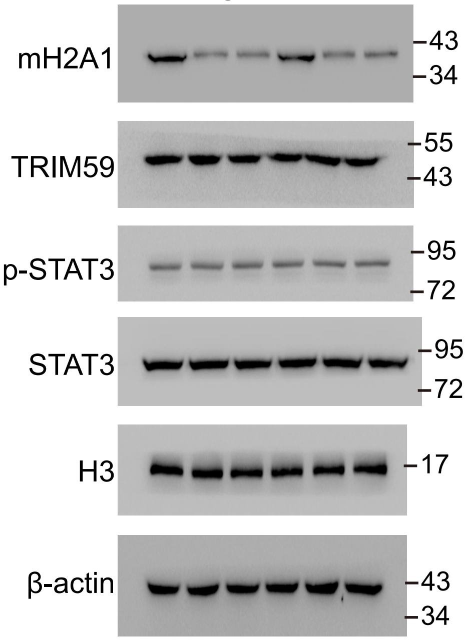

Figure 7I

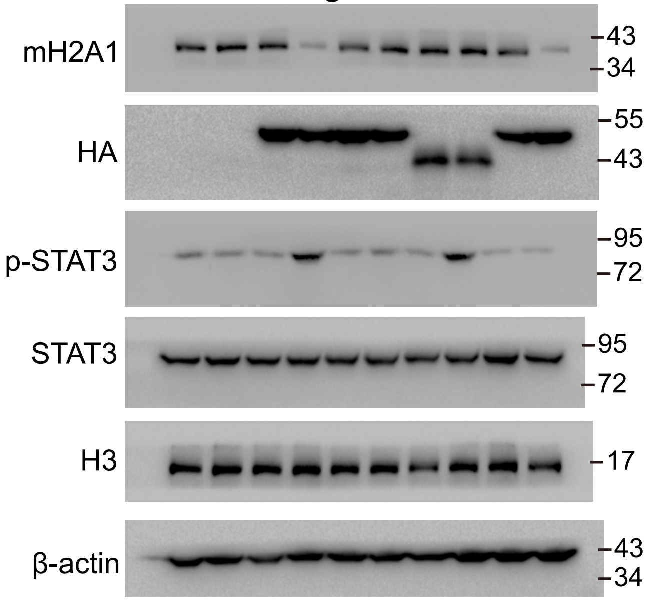

Figure 8A

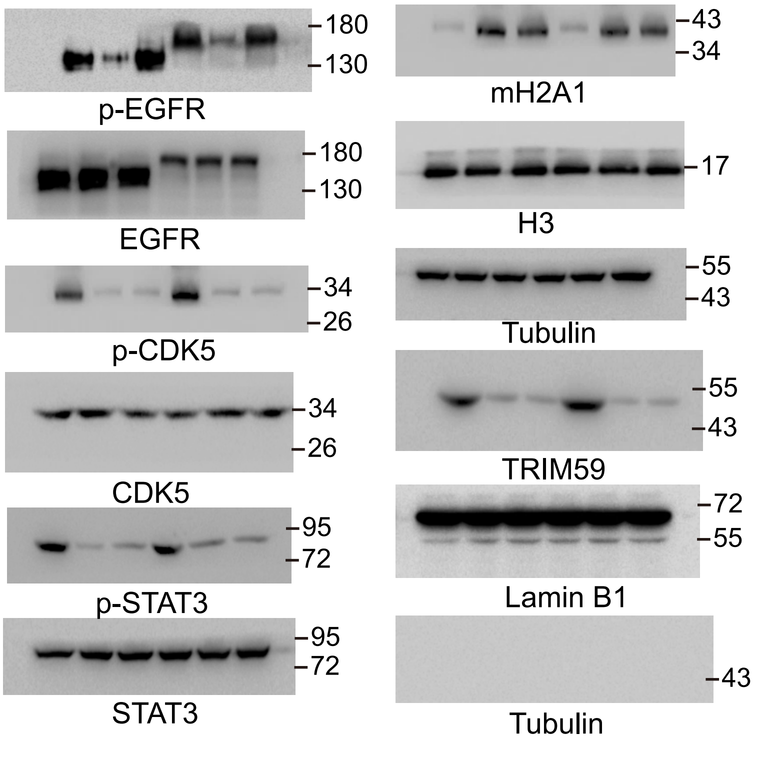

Figure 8D

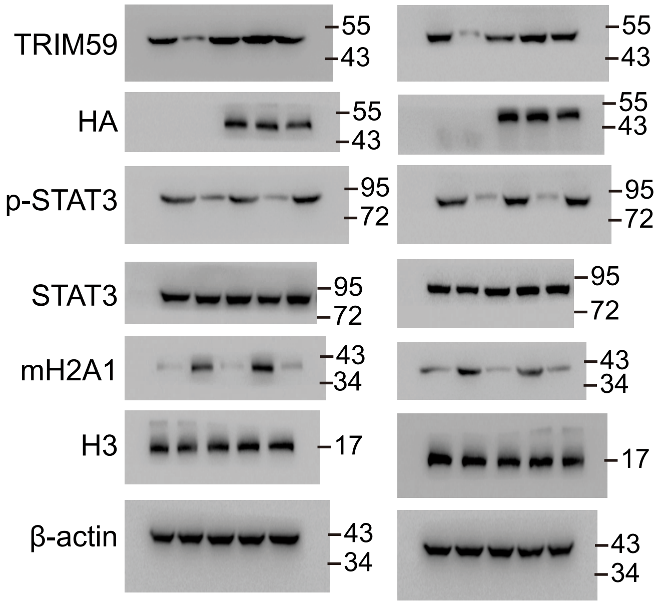

Figure 8J

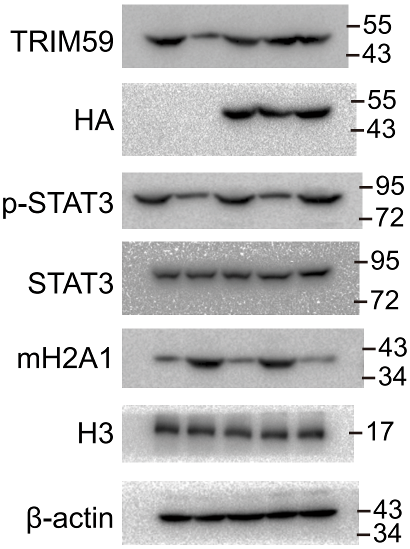

Figure S1B

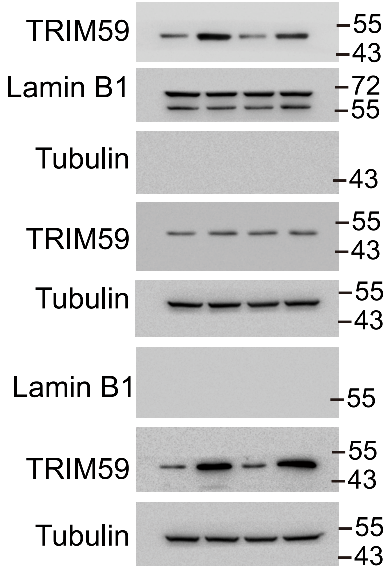

Figure S2A

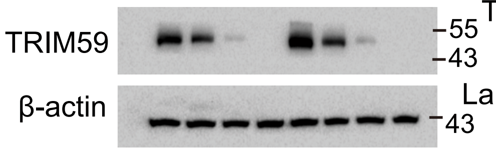

Figure S3

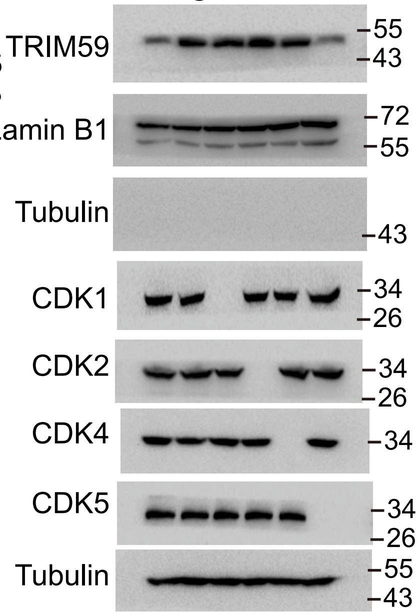

Figure S4B

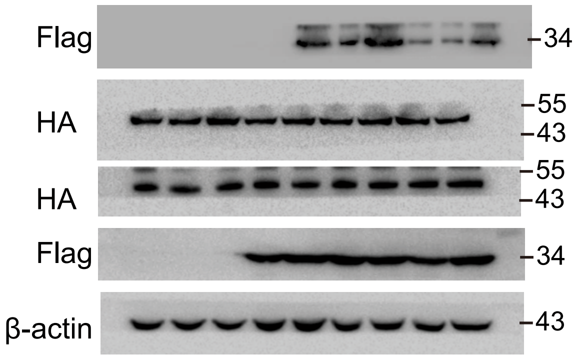

Figure S4A

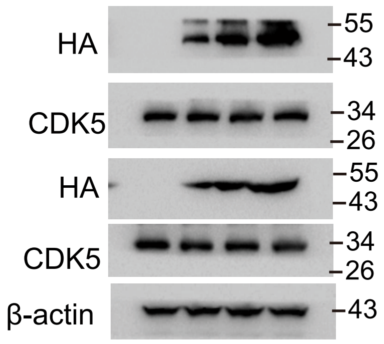

Figure S5B

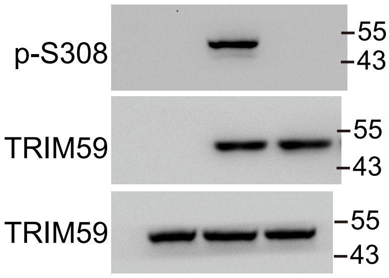

Figure S6

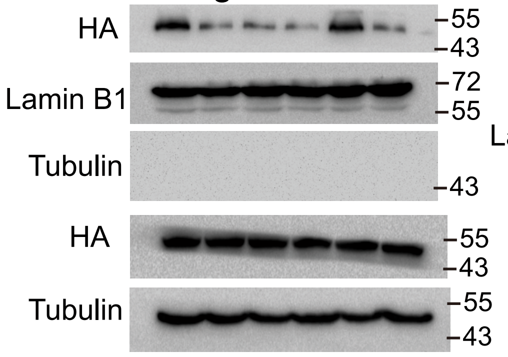

Figure S7

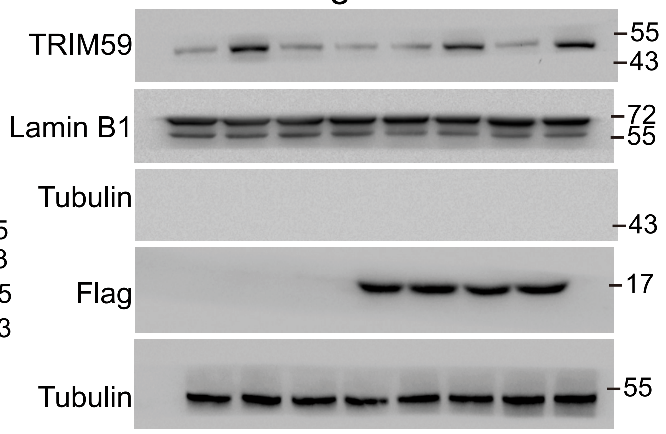

Figure S9A

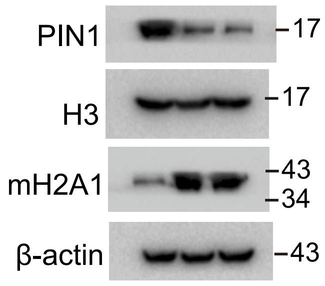

Figure S9B

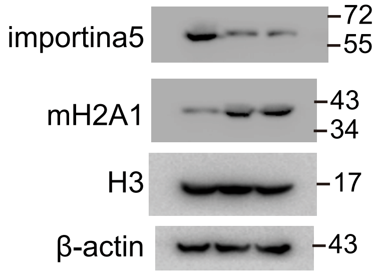

Figure S10B

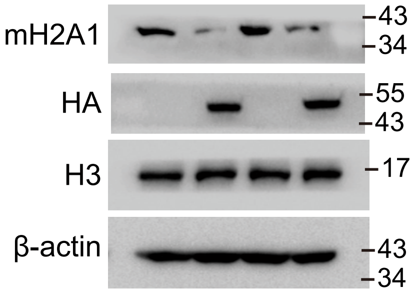

Figure S10C

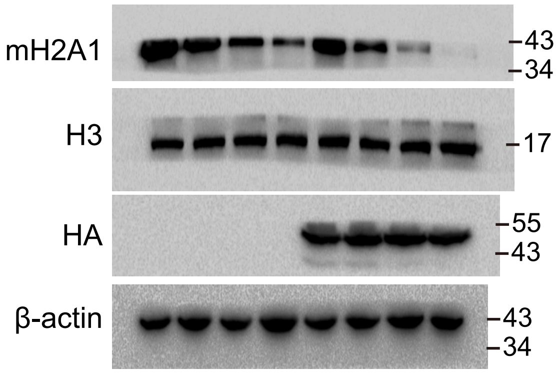

Figure S10E

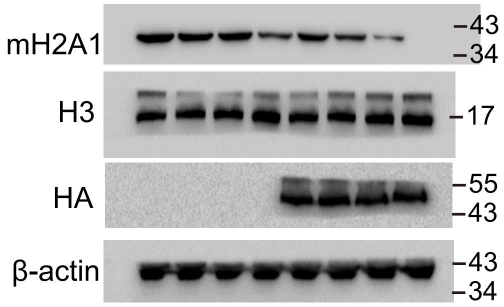

Figure S12

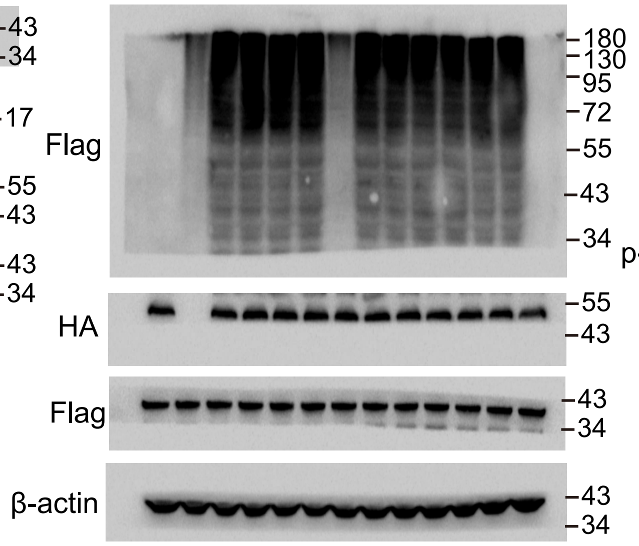

Figure S13

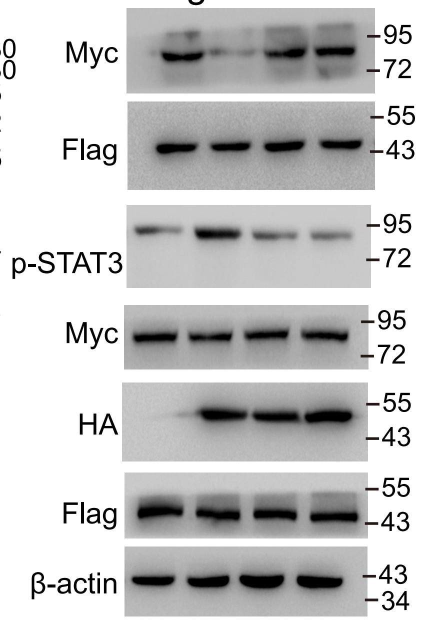

Figure S11

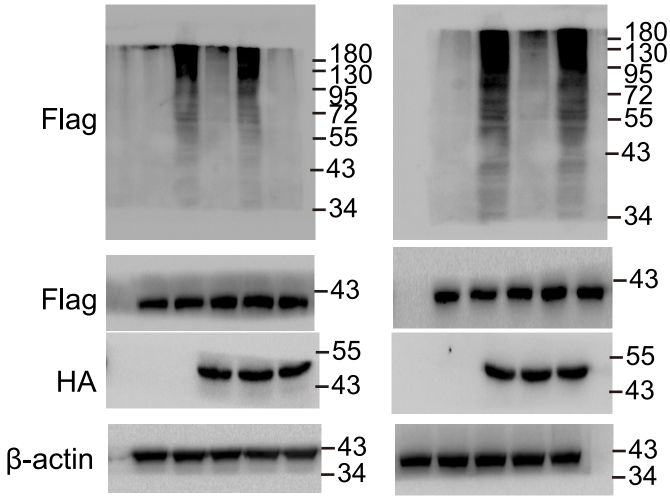

Figure S14A

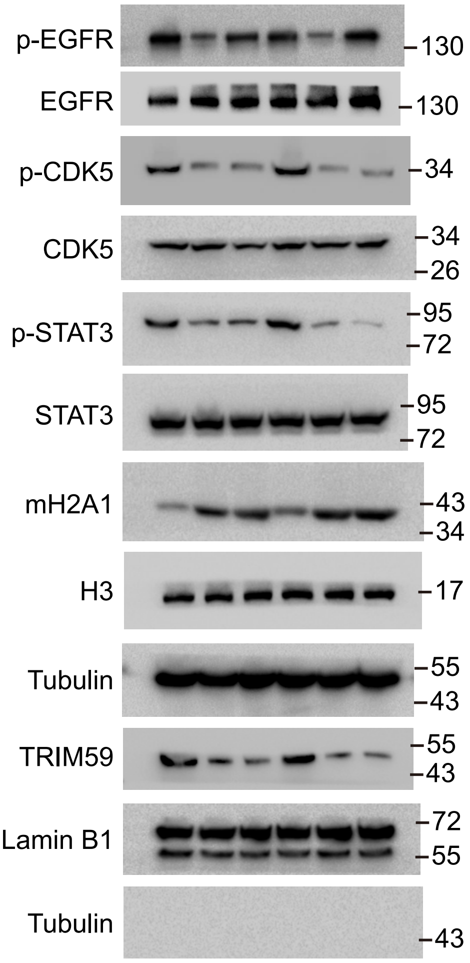

Figure S14C

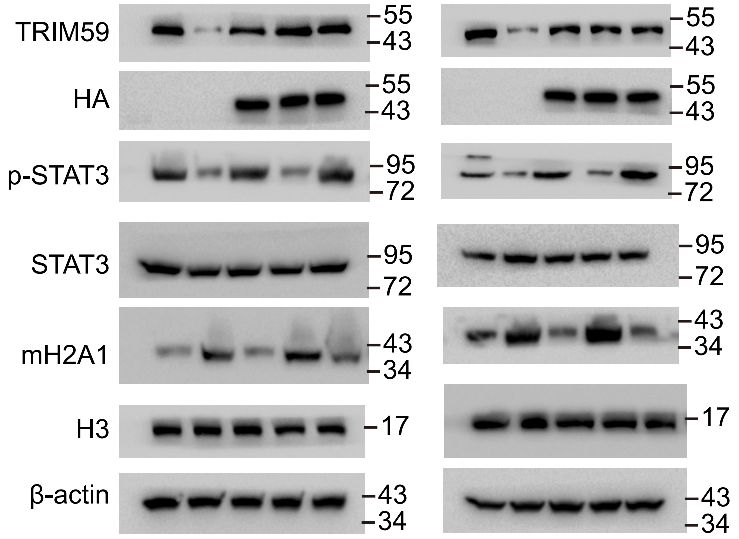

Supplementary Figure 16 Full unedited gels
